# The Herbal Combination of *Radix astragali*, *Radix angelicae sinensis*, and *Caulis lonicerae* Regulates the Functions of Type 2 Innate Lymphocytes and Macrophages Contributing to the Resolution of Collagen-Induced Arthritis

**DOI:** 10.3389/fphar.2022.964559

**Published:** 2022-07-19

**Authors:** Guiyu Feng, Dongyang Li, Juan Liu, Song Sun, Pingxin Zhang, Wei Liu, Yingkai Zhang, Boyang Meng, Jinyu Li, Limin Chai

**Affiliations:** ^1^ Key Laboratory of Chinese Internal Medicine of Ministry of Education and Beijing, Dongzhimen Hospital, Beijing University of Chinese Medicine, Beijing, China; ^2^ Department of Pharmacy, The Third Affiliated Hospital of Beijing University of Chinese Medicine, Beijing, China; ^3^ Department of Orthopedic, Dongzhimen Hospital, Beijing University of Chinese Medicine, Beijing, China

**Keywords:** *R*. *astragali*, *R*. *angelicae sinensis*, *C*. *lonicerae*, type 2 innate lymphocytes, inflammation resolution, collagen-induced arthritis

## Abstract

Type 2 innate lymphocytes (ILC2s), promoting inflammation resolution, was a potential target for rheumatoid arthritis (RA) treatment. Our previous studies confirmed that *R*. *astragali* and *R*. *angelicae sinensis* could intervene in immunologic balance of T lymphocytes. *C*. *lonicerae* also have anti-inflammatory therapeutic effects. In this study, the possible molecular mechanisms of the combination of these three herbs for the functions of ILC2s and macrophages contributing to the resolution of collagen-induced arthritis (CIA) were studied. Therefore, we used *R*. *astragali*, *R*. *angelicae sinensis*, and *C*. *lonicerae* as treatment. The synovial inflammation and articular cartilage destruction were alleviated after herbal treatment. The percentages of ILC2s and Tregs increased significantly. The differentiation of Th17 cells and the secretion of IL-17 and IFN-γ significantly decreased. In addition, treatment by the combination of these three herbs could increase the level of anti-inflammatory cytokine IL-4 secreted, active the STAT6 signaling pathway, and then contribute to the transformation of M1 macrophages to M2 phenotype. The combination of the three herbs could promote inflammation resolution of synovial tissue by regulating ILC2s immune response network. The synergistic effects of three drugs were superior to the combination of *R*. *astragali* and *R*. *angelicae sinensis* or *C*. *lonicerae* alone.

## Introduction

Rheumatoid arthritis (RA) is a chronic autoimmune disease characterized by symmetrical, progressive, and erosive arthritis (Smolen et al., 2016). The key pathological changes of RA are the continuous infiltration of immune cells into the synovial tissues, leading to the occurrence of autoimmune inflammation in the synovial tissues. Effector T-cells and B cells form a complex immune network in the damaged tissues, promote the production of inflammatory cytokines, stimulate the inflammatory proliferation of fibroblast-like synoviocytes (FLSs) in synovial tissues of inflamed joints, and secrete inflammatory cytokines, induce autoimmune inflammation and lead to the damage of articular cartilage (McInnes and Schett, 2007; Schett et al., 2013; McInnes and Schett, 2017). Previous studies have confirmed that macrophages polarize into a pro-inflammatory “M1” phenotype in RA, increases the production of pro-inflammatory mediators and decreases the secretion of anti-inflammatory cytokines, such as interleukin (IL)-4, IL-13, and IL-10 (Murray et al., 2014). The Janus tyrosine kinase/signal transducer and activator of transcription (JAK-STAT) signaling pathway plays an important role in the pathogenesis of RA synovitis. Anti-arthritis by interfering with JAK/STAT pathway has been reported, further establishing the key role of JAK/STAT pathway activation in RA (Banerjee et al., 2017; Malemud, 2018).

Current treatment regimens for RA mainly focus on targeting the production of pro-inflammatory cytokines and the activation of autoimmune inflammation (Smolen et al., 2020). Neutralizing antibodies, targeting to tumor necrosis factor (TNF), IL-6 and Janus tyrosine kinase (JAK), can effectively inhibit the process of synovitis and articular cartilage injury. This therapeutic has been widely used for RA treatment (Schmid and Neri, 2019; Silvagni et al., 2020). Nevertheless, immunosuppressive therapy often brings the risk of secondary infection because of excessive suppression (Bongartz et al., 2006; Singh et al., 2015). At present, the therapeutic goal of RA is focus on induce inflammation remission. Innate lymphoid cells (ILCs) play complex roles throughout the duration of immune responses (McKenzie et al., 2014). Activation of different types of ILCs through different immune response networks can aggravate or slow down the inflammatory and pathological process of autoimmune diseases (Klose and Artis, 2016). Several researches showed that ILC2s could attenuate inflammatory arthritis and protect from bone destruction (Omata et al., 2018). ILC2s should be a potential therapeutic target for the novel treatment strategies in RA.

Chinese herbal treatment has promising immunomodulatory effects *via* multiple targets for RA treatment (Ma et al., 2013). *R*. *astragali*, *R*. *angelicae ainensis*, and *C*. *lonicerae* have been widely used in clinical practice for a long time and proved to be safe and effective. Pharmacological researches indicated that both *R*. *astragali* and *R*. *angelicae sinensis* have therapeutical effect on RA, including regulating the immune system (Yang et al., 2006; Chen et al., 2020), anti-inflammation (Magdalou et al., 2015; Wang et al., 2016), anti-oxidation (Zhang et al., 2010; Shahzad et al., 2016) and promoting bone formation (Yang et al., 2014). Pharmacological studies confirmed that *C*. *lonicerae* had broad spectrum of pharmacological activities, such as anti-inflammation (Su et al., 2021), anti-oxidation (Li et al., 2020), and anti-angiogenic (Yoo et al., 2008). In our previous studies, we proved that chlorogenic acid and Luteolin, the main components of *C*. *lonicerae*, could inhibit inflammatory proliferation of FLSs induced by IL-1β and IL-6 through activating FLSs apoptosis (Lou et al., 2015; Lou et al., 2016). Furthermore, herbal formula (Xianfanghuomingyin, XFHM), *C*. *lonicerae* as the main ingredient, has been proved which had therapic effect on alleviating cartilage destruction and pannus formation in collagen-induced arthritis (CIA) mice (Nie et al., 2016; Li et al., 2017). Therefore, we suggest that the combination of *R*. *astragali*, *R*. *angelicae sinensis* and *C*. *lonicerae* can promote the resolution of synovial inflammation and inhibit the subsequent destruction of cartilage.

In this study, we used CIA mice as animal model. Leflunomide (LEF) was used as a positive control medicine. We want to investigate the mechanism of the combination of *R*. *astragali*, *R*. *angelicae sinensis*, and *C*. *lonicerae* for inhibiting abnormal immune response and promoting inflammation resolution in synovial tissues of CIA mice through regulating the functions of ILC2s and macrophages.

## Material and Methods

### Preparation of the Herbal Medicine and HPLC-ESI/MS^n^ Analysis

A total of 100 g of raw herbal pieces, including *R*. *astragali* (origin: Inner Mongolia, China, 80 g), *R*. *angelicae sinensis* (origin: Gansu, China, 20 g), were soaked in water for 30 min and then decocted to an extract solution (500 mg/ml). *C*. *lonicerae* (origin: Henan, China, 60 g), were soaked in water for 30 min and then decocted to an extract solution (300 mg/ml). A total of 160 g of raw herbal pieces, including *R*. *astragali* (origin: Inner Mongolia, China, 80 g), *R*. *angelicae sinensis* (origin: Gansu, China, 20 g), *C*. *lonicerae* (origin: Henan, China, 60 g), were soaked in water for 30 min, and then decocted to an extract solution (800 mg/ml). The herbs were provided by the Pharmacy Department of Dongzhimen Hospital of Beijing University of Chinese Medicine.

The constituents of *R*. *astragali*, *R*. *angelicae sinensis*, and *C*. *lonicerae* were detected using HPLC-ESI/MS^n^. The specific detection methods were based on our previous description (Nie et al., 2016; Li et al., 2017). The Peak View Software™ 2.2 (SCIEX, Foster City, CA, United States) was used to analyze the data by signal intensity and retention time.

### Collagen-Induced Arthritis Induction in DAB1/J Mice and Drug Treatment

DBA1/J male mice (*n* = 8 per group, 7–8 weeks old, 18 ± 2 g weight) were purchased from Beijing Vital River Laboratory Animal Technology Co., Ltd. (Beijing, China). Animal care and use were in accordance with institutional guidelines. All animal experiments were approved by the Animal Ethics Committee of Beijing University of Chinese Medicine.

Mice, except the control group, were immunized intradermally with 100 μg of bovine type II collagen (CII) (Batch number 190209, Chondrex, Seattle, WA, United States) emulsified with an equal volume of complete Freund’s adjuvant (CFA) (Batch number 190214, Chondrex, Seattle, WA, United States). Injection was performed on the base of the tail and back. Mice were boosted by intradermal injection with 100 μg of CII emulsified with incomplete Freund’s adjuvant (IFA) (Batch number 190269, Chondrex, Seattle, WA, United States) on the 21st day after first immunization. The model was completed on the 28th day after the first immunization (incidence rate 94.4%). Then the sagittal plane radius of the hind limb was measured every 7 days, the thickness of metatarsal joint of the hind limb was also measured by vernier caliper with 50-divisions.

Drug treatment began at 7 days after booster immunization and lasted for 28 days. Mice were divided into six groups as follows: control group, mice fed the control diet, and orally given sterile saline; model group, mice fed the same as the control group; LEF group, mice fed the control diet, and orally daily given 3 mg/kg/d LEF (No. H20080047, Suzhou Changzheng-Xinkai Pharmaceutical Co., Ltd., China) for 28 days; *R*. *astragali* and *R*. *angelicae sinensis* combination group (R + A group), mice fed the control diet and orally given 7.5 g/kg/d the two-herb combination daily for 28 days; *C*. *lonicerae* group (C group), mice fed the control diet and orally given 4.5 g/kg/d herb daily for 28 days; *R*. *astragali*, *R*. *angelicae sinensis*, and *C*. *lonicerae* combination group (R + A + C group), mice fed the control diet and orally given 12 g/kg/d the three-herb combination daily for 28 days. The mice were sacrificed on the 29th day after treatment.

### Clinical Scores of Collagen-Induced Arthritis

Clinical scores of CIA were monitored every 7 days after booster immunization. Scores for swelling of paws were classified as follows: as 0 (normal joints), 1 (swelling in one digit or joint inflammation), 2 (swelling in two or three digits, or slight paw swelling), 3 (swelling in more than four digits and moderate swelling in the entire paw), and 4 (severe swelling and deformation of the paw). The clinical score is the sum of the scores of all four paws of each mouse, with a maximum score of 16.

### Histology

The left legs and hind paws of mice were removed, fixed overnight in 4% paraformaldehyde at 4°C. After fixation, skin, and muscle were completely removed, and the plantar joints of mice were dissected, and immersed in 10% EDTA for sufficient decalcification. Then embedded in paraffin and sectioned at 5 μm thickness. The paraffin sections were deparaffinized with xylene and rehydrated with gradient ethanol, then stained with hematoxylin-eosin (HE), safranin O-fast green (Safranin O) or tartrate-resistant acid phosphatase (TRAP), the specimens were observed and photographed by a light microscope (DM RAS2 Leica, Solms, Germany). Histopathological changes in synovial inflammation, cartilage destruction, and bone erosion were assessed according to previously reported methods (Feng et al., 2019). The scores of Loss of safranin O staining (Rauber et al., 2017) were defined as follows: no loss (0 score); slight loss (1 score); moderate loss (2 score); severe loss (3 score); completely loss (4 score).

### Immunofluorescence Staining

After deparaffinization and rehydration through standard protocols, the paraffin sections were washed by PBS. Then sections were alternately bathed in boiling sodium citrate buffer for 20 min. After returning to room temperature, sections were washed. The membrane was permeated in 0.3% PBST (100 ml PBS, 0.3 ml Triton X-100) for 20 min. Sections were washed and then blocked by donkey serum at 37°C for 30 min. Sections were incubated by primary antibodies overnight at 4°C, including anti-F4/80 antibody (1:100, abcam), anti-INOS antibody (1/100, abcam), and anti-CD206 antibody (1:100, R&D). After incubtion the sections were restored to room temperature, washed by PBS, and then incubated by secondary antibodies, including donkey secondary antibodies to rat (1:2,000, Alexa Fluor^®^488, abcam), donkey secondary antibodies to rabbit (1:2,000, Alexa Fluor^®^647, abcam), and donkey secondary antibodies to goat (1:2,000, Alexa Fluor^®^555, abcam). After antibody incubation, sections were washed and finally sealed in a sealant containing DAPI. Images were acquired through a Leica confocal laser scanning microscopy (Leica).

### Flow Cytometry Analysis

Lymph nodes and synovial tissues were respectively removed, diced, and expressed through a 70 μm Nylon mesh. All isolated lymphocytes and synovial cells were re-suspended as a single cell suspension. To determine the percentage of Th17 cells, cells derived from synovial tissues were washed and stained with FITC anti-mouse CD4 Antibody (Biolegend). Following CD4 staining, cells were blocked, fixed, and permeabilized through a Fixation/Permeabilization kit according to manufacturers’ instructions (BD Bioscience), and then further stained with PE/Cyanine7 anti-mouse IL-17A Antibody (Biolegend). To determine the percentage of Treg cells, cells were washed and stained with FITC anti-mouse CD4 Antibody (Biolegend) and PE anti-mouse CD25 Antibody (Biolegend). Following CD4 and CD25 staining, cells were blocked, fixed, and permeabilized using a Fixation/Permeabilization kit according to manufacturers’ instructions (BD Bioscience), and stained with FOXP3 Monoclonal Antibody (eBioscience). To determine the percentage of Memory T-cells, Effector T-cells, and Naïve T-cells, cells were washed and stained with PE anti-mouse/human CD44 Antibody (Biolegend) and CD62L Monoclonal Antibody (eBioscience). To determine the percentage of ILC2s, cells derived from lymph nodes were washed and stained with Pacific Blue™ anti-mouse Lineage Cocktail with Isotype Ctrl (Biolegend), PE anti-mouse/human KLRG1 (MAFA) Antibody (Biolegend), FITC anti-mouse CD127 (IL-7Rα) Antibody (Biolegend), and anti-human/mouse/rat CD278 (ICOS) Antibody (Miltenyi). Flow cytometry was performed by a FACS Calibur cytometer and analyzed using CellQuest software (Beckman Coulter, CA, United States).

### Enzyme-Linked Immunosorbent Assay

Twenty-four hours after the last administration, 0.8 ml of peripheral blood was collected from each mouse by eyeball extirpation. Sera were isolated by centrifuging at 3,000 rpm and 4°C for 10 min. The ELISA kit (eBioscience) was used to quantitate the contents of IFN-γ, IL-17, and IL-4 in serums following the instructions strictly.

### Western Blotting Analysis

Proteins for western blotting analysis were extracted from the synovial tissues and lysed by RIPA Lysis Buffer (Boster, Wuhan, China) with protease and phosphatase inhibitors (Boster, Wuhan, China). The protein concentration was quantified preliminarily with the BCA kit (Boster, Wuhan, China). The total proteins were separated using 7.5% SDS-PAGE (Epizyme, Shanghai, China), and then transferred onto nitrocellulose membranes. The membranes were blocked by TBST containing 5% skimmed milk for 1 h at room temperature. The primary antibodies including JAK2 polyclonal antibody (1:1,000, Thermo Fisher), phospho-JAK2 polyclonal antibody (1/1,000, Thermo Fisher), JAK3 polyclonal antibody (1:1,000, Thermo Fisher), phospho-JAK3 polyclonal antibody (1:1,000, Thermo Fisher), STAT6 polyclonal antibody (1:1,000, Thermo Fisher), phospho-STAT6 polyclonal antibody (1:1,000, Thermo Fisher) were incubated overnight at 4°C. Next, the membranes were incubated with HRP-conjugated secondary antibody (1/8,000, Boster, Wuhan, China) for 1 h, and then treated with ECL chemiluminescence reagents. Densitometry plots showing protein expression were analyzed by ImageJ (Bethesda, United States). Densitometry plots of the protein expression levels were normalized to GAPDH (1/2,000, Thermo Fisher).

### Statistical Analysis

All data are presented as the mean ± standard deviation (SD). Statistical analyses were performed using SPSS 20.0. One-way analysis of variance (ANOVA) followed by the Tukey-Kramer test for multiple comparisons was used to compare with the treatment groups. *p* < 0.05 was considered statistically significant.

## Results

### Identification of Chemical Constituents in Three Herbs by HPLCESI/MS^n^

**FIGURE 1:**
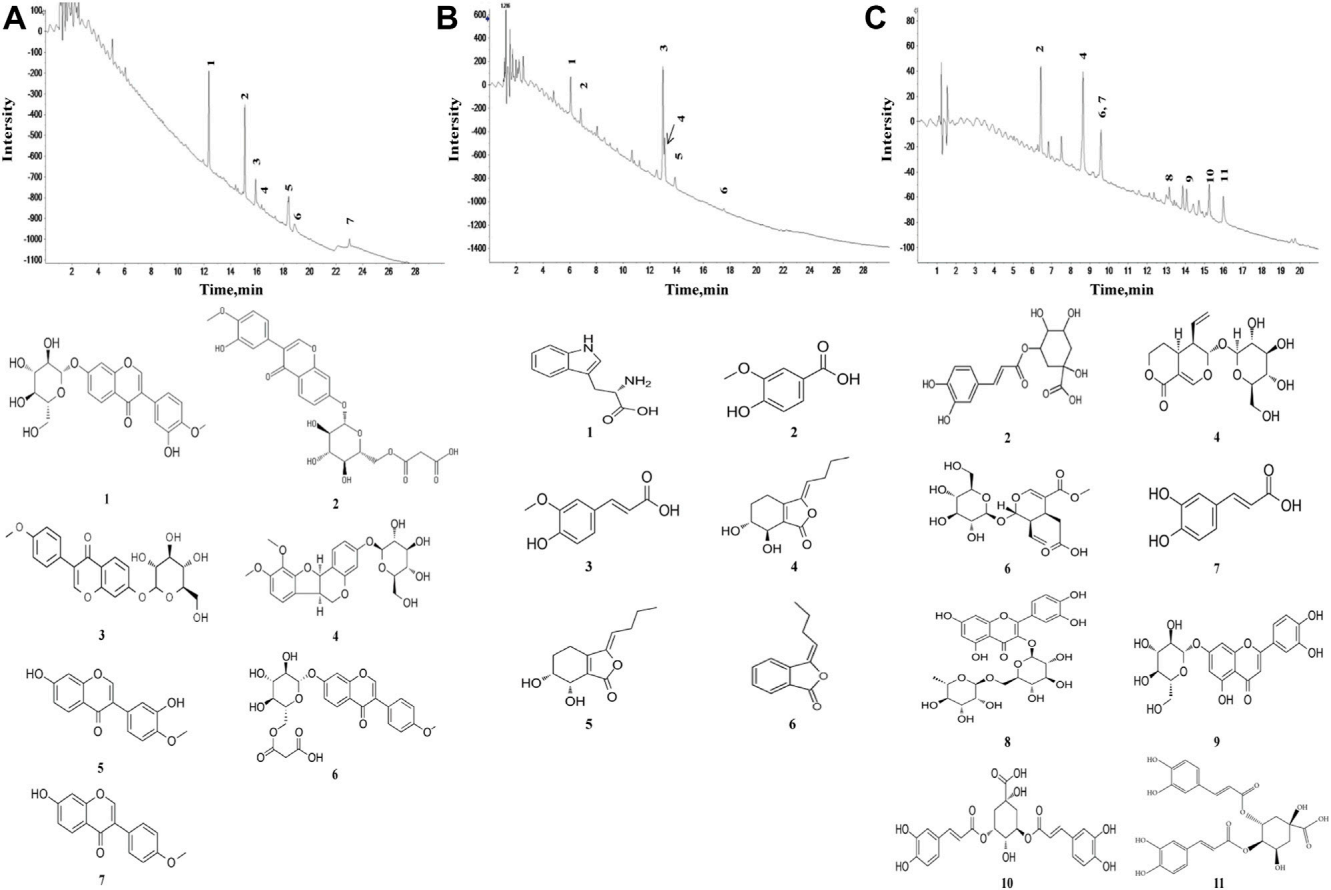
Characteristics of pure compounds from the aqueous extract of *R*. *astragali, R*. *angelicae sinensis*, and *C*. *lonicerae*. The abscissa represents the retention time, and the ordinate represents the chromatographic peak intensity. **(A)** HPLC-ESI/MS^n^ total ion chromatogram of *R*. *astragali* and the molecular formulas of identified components. **(B)** HPLC-ESI/MS^n^ total ion chromatogram of *R*. *angelicae sinensis* and the molecular formulas of identified components. **(C)** HPLC-ESI/MS^n^ total ion chromatogram of *C*. *lonicerae* and the molecular formulas of identified components.

Seven constituents of *R*. *astragali*, six constituents of *R*. *angelicae sinensis*, and eleven constituents of *C*. *lonicerae* (Figure 1) were identified based on the target peaks. The identified compounds of these herbs are shown in Tables 1–3.

**TABLE 1:** Chemical components identified from *R*. *astragali* by HPLC-ESI/MS^n^.

Peak	t_R_	Identification
1	12.437	Calycosin-7-*O*-β-D-glucoside
2	15.161	Calycosin-7-*O*-β-D-glucoside-6″-*O*-malonate
3	15.979	Ononin
4	16.624	(6a*R*,-11a*R*)-3-Hydroxy-9,10-dimethoxypterocarpan-3-*O*-β-D-glucoside
5	18.416	Calycosin
6	18.498	Formononetin-7-*O*-β-D-glucoside-6″-*O*-malonate
7	23.087	Formononetin

**TABLE 2:** Chemical components identified from *R*. *angelicae sinensis* by HPLC-ESI/MS^n^.

Peak	t_R_ (min)	Identification
1	6.14	L-tryptophan
2	6.909	Vanillic acid
3	13.06	Ferulic acid
4	13.2	Senkyunolide I
5	13.973	Senkyunolide H
6	17.623	E-Butylidenephthalide

**TABLE 3:** Chemical components identified from *C*. *lonicerae* by HPLC-ESI/MS^n^.

Peak	t_R_ (min)	Identification
1	5.899	Loganin acid
2	6.509	3-O-caffeoylquinic acid
3	8.241	Loganin
4	8.515	Sweroside
5	8.715	4-O-caffeoylquinic acid
6	9.602	Secoxyloganin
7	9.665	Caffeic acid
8	13.38	Rutin
9	14.146	Cynaroside
10	15.33	3,5-O-dicaffeoylquinic acid
11	16.071	3,4-O-dicaffeoylquinic acid

### The Combination of Herbs Relieved the Joints Swelling of Collagen-Induced Arthritis Mice

**FIGURE 2:**
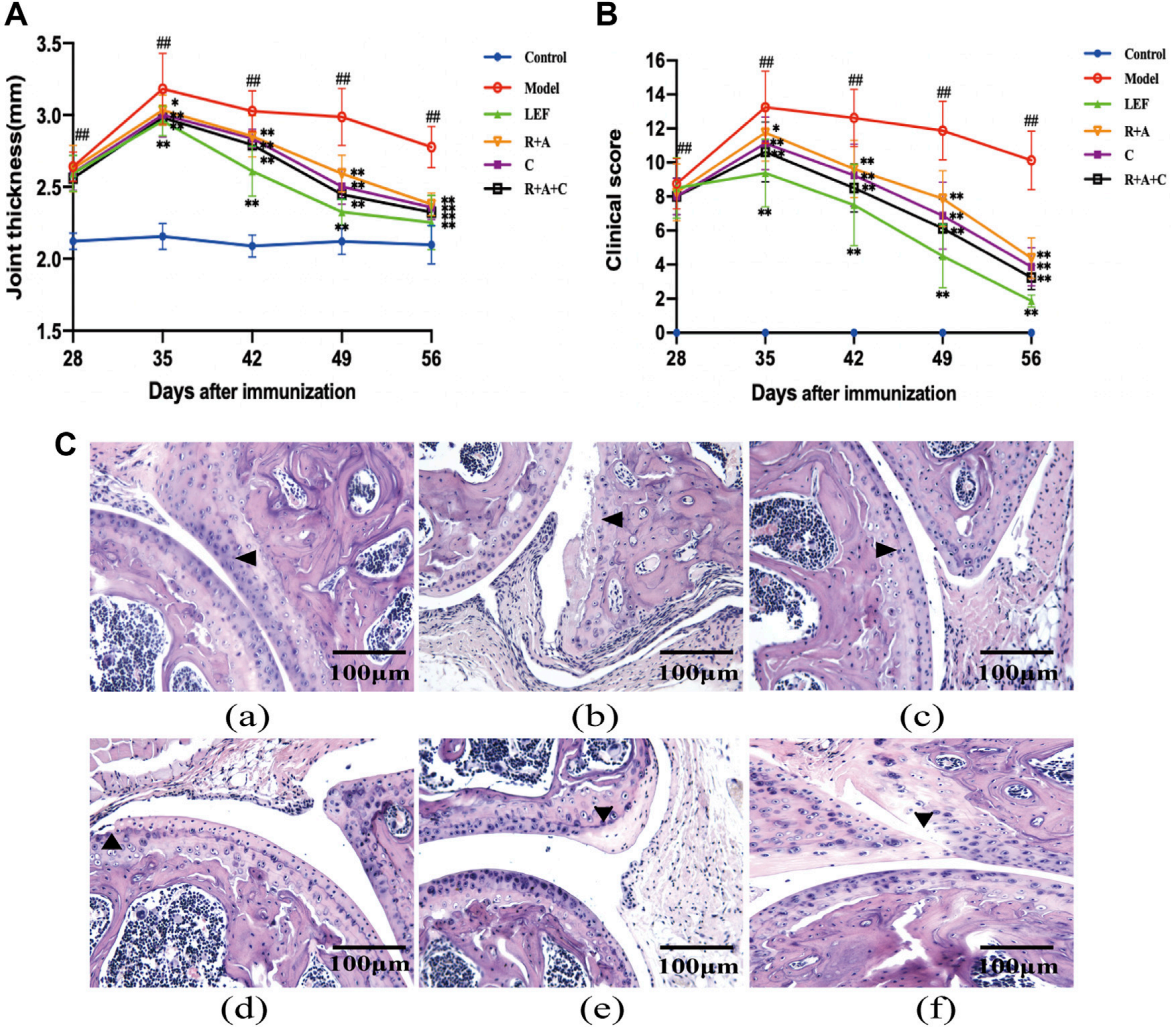
The combination of three herbs reduced the severity of joint swelling in CIA mice. **(A)** Joint thickness of control and CIA mice. **(B)** Clinical scores of control and CIA mice. **(C)** Hematoxylin-eosin (HE) staining of synovial tissue of joint (magnification ×200). The triangle indicates articular cartilage. **(a)**, control group; **(b)**, model group; **(c)**, LEF group; **(d)**, *R*. *astragali* + *R*. *angelicae sinensis* (R + A) group; **(e)**, *C*. *lonicerae* (C) group; and **(f)**, *R*. *astragali* + *R*. *angelicae sinensis* + *C*. *lonicerae* (R + A + C) group. All data are shown as mean ± SD (*n* = 8); ^#^ *p* < 0.05, ^##^ *p* < 0.01, compared with the normal group; *p < 0.05, **p < 0.01, compared with the model group.

Drug intervention was initiated on the 28th day after immunization and lasted 28 days. The thickness of the bilateral plantar joints and clinical scores were detected and measured weekly. As shown in Figure 2, the R + A, C, R + A + C, and LEF treatment significantly decreased the thickness of the bilateral plantar joints (*p* < 0.01, Figure 2A) and significantly reduced the clinical scores (*p* < 0.01, Figure 2B) on the 56th day. The curative effect of R + A + C group was better than those of other herbal groups.

Histopathological lesions in the joints were observed through HE staining (Figure 2C). The synovial hyperplasia, cartilage destruction, and bone erosion in the joints were assessed. As shown in Figure 2C, the histopathological lesions of CIA mice were alleviated by treatment with R + A, C, R + A + C, and LEF. The ameliorative effect of R + A + C group was better than those of R + A and C groups.

### The Combination of Three Herbs Repaired the Bone Destruction in the Joints of Collagen-Induced Arthritis Mice

**FIGURE 3:**
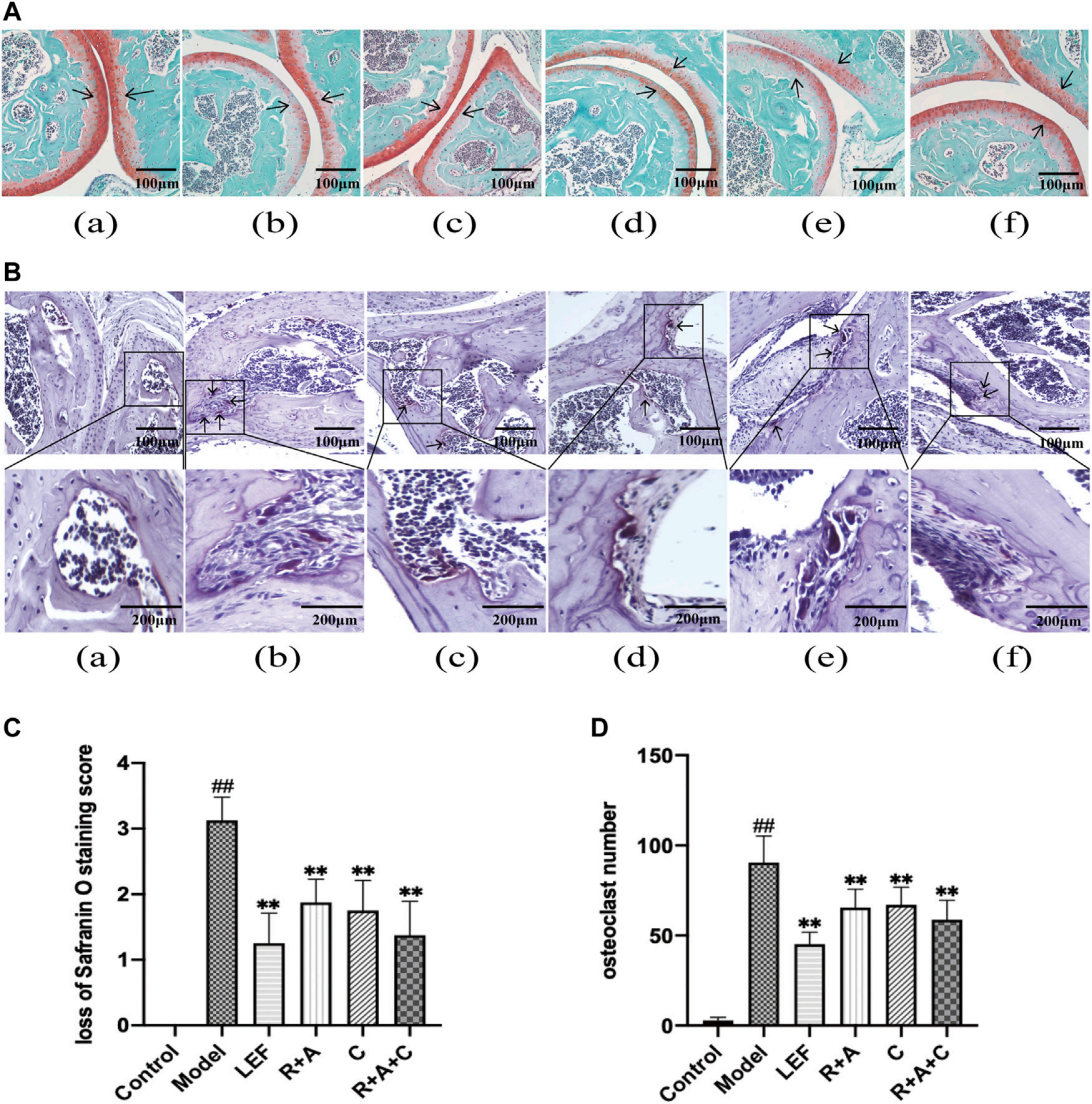
The combination of three herbs improved bone destruction in CIA mice. **(A)** Saffron O staining of articular tissue (magnification ×200). The arrow indicates articular cartilage. **(B)** Tartrate-resistant acid phosphatase (TRAP) staining of articular tissue (magnification of the first row ×200). The arrow points to osteoclasts. **(C)** score of loss of safranin O staining. **(D)** number of osteoclasts. **(a)**, control group; **(b)**, model group; **(c)**, LEF group; **(d)**, *R*. *astragali* + *R*. *angelicae sinensis* (R + A) group; **(e)**, *C*. *lonicerae* (C) group; and **(f)**, *R*. *astragali* + *R*. *angelicae sinensis* + *C*. *lonicerae* (R + A + C) group. All data are shown as mean ± SD (*n* = 8); ^#^ *p* < 0.05, ^##^ *p* < 0.01, compared with the normal group; *p < 0.05 **p < 0.01, compared with the model group. The scale bar corresponds to 100 µm or 200 µm.

Safranin O staining can directly reflect the structure of articular cartilage and subchondral bone. After staining, the cartilage appears red. There were also differences in Safranin O staining observed among the groups (Figure 3A). The intensity of Safranin O staining in CIA mice decreased significantly compared with the control mice. As shown in Figure 3C, after treated by LEF, R + A, C, and R + A + C, the loss scores of Safranin O staining decreased. Thus, herbal treatment increased the staining significantly, and the enhancement effect of R + A + C group was better than those of other herbal treatment groups.

As shown in TRAP staining (Figure 3B), the purplish red osteoclasts were mostly expressed on the bone surface. The significant differences were observed between normal mice and CIA mice. R + A, C, and R + A + C treatment reduced the abnormal expression of osteoclasts (Figure 3D).

### The Proliferation and Differentiation of CD4^+^ T-Cells Were Regulated by Herbal Treatment

**FIGURE 4:**
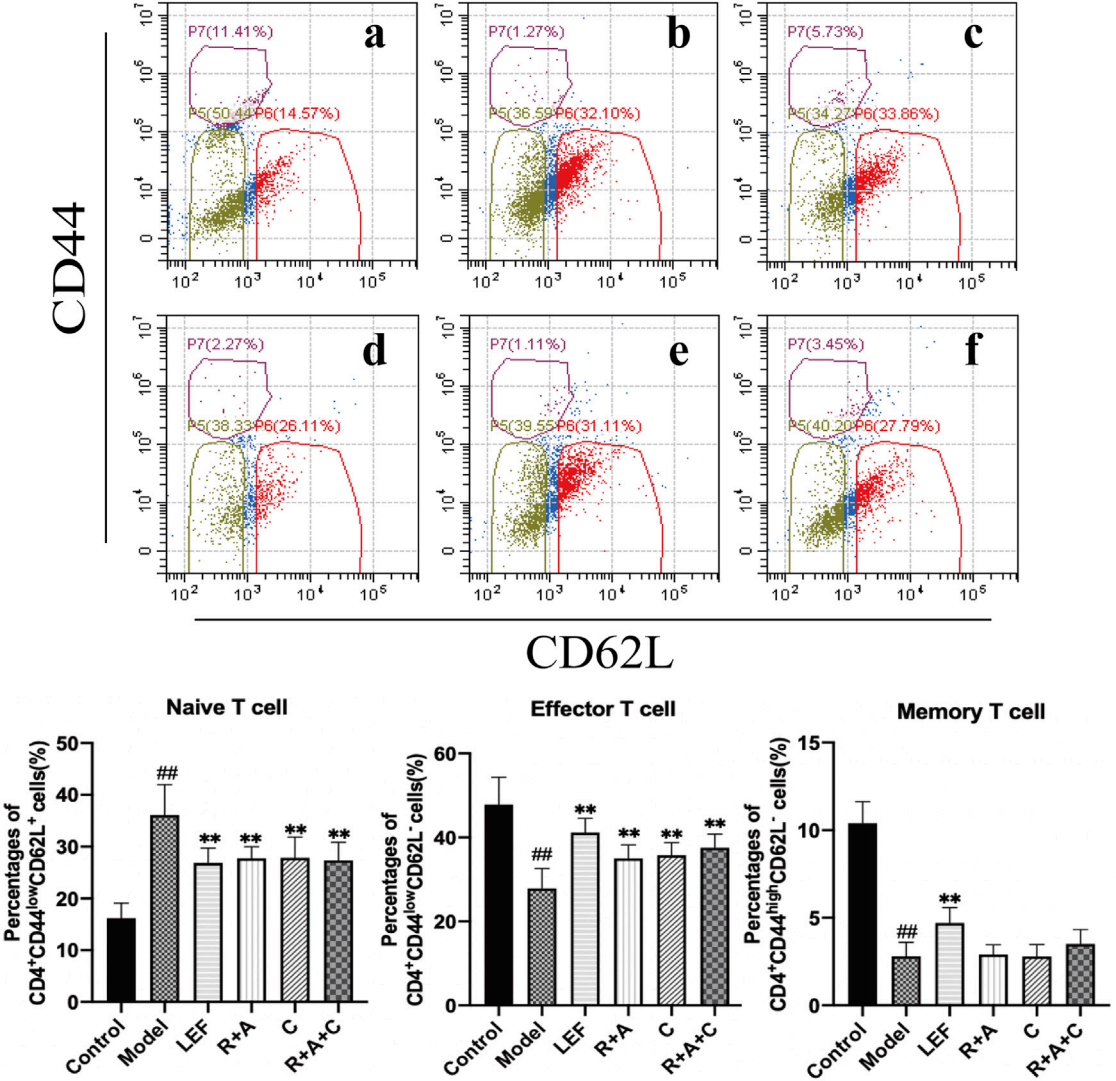
The percentages of CD4^+^CD44^High^CD62L^−^ T-cells, CD4^+^CD44^Low^CD62L^−^ T-cells and CD4^+^CD44^Low^CD62L^+^ T-cells. **(a)**, control group; **(b)**, model group; **(c)**, LEF group; **(d)**, *R*. *astragali* + *R*. *angelicae sinensis* (R + A) group; **(e)**, *C*. *lonicerae* (C) group; and **(f)**, *R*. *astragali* + *R*. *angelicae sinensis* + *C*. *lonicerae* (R + A + C) group. Results are presented in a bar chart. Data are presented as mean ± SD (*n* = 8); ^#^ *p* < 0.05, ^##^ *p* < 0.01, compared with the normal group; *p < 0.05, **p < 0.01, compared with the model group.

CD4^+^ T-cells coordinate diverse immune responses to deal with various disease-causing pathogens. Activated naïve CD4^+^ T-cells differentiate into several subsets of effector cells that have different functions, including Th17 and Treg cells. The final fate is primarily determined by the external milieu (e.g., cytokines) present during activation (Lee, 2018). As shown in Figure 4, R + A, C, and R + A + C treatment decreased the level of naive (CD4^+^CD44^Low^CD62L^+^) T-cells (*p* < 0.01) significantly and increased the level of effector (CD4^+^CD44^Low^CD62L^−^) T-cells in CIA mice after treatment, whereas the decreasing effect on the memory (CD4^+^CD44^High^CD62L^−^) T-cells was not obvious. The intervention effect of R + A + C group was better than those of other herbal treatment groups.

### The Herbal Treatments Intervened in the Proliferation and Differentiation of Th17 and Tregs

**FIGURE 5:**
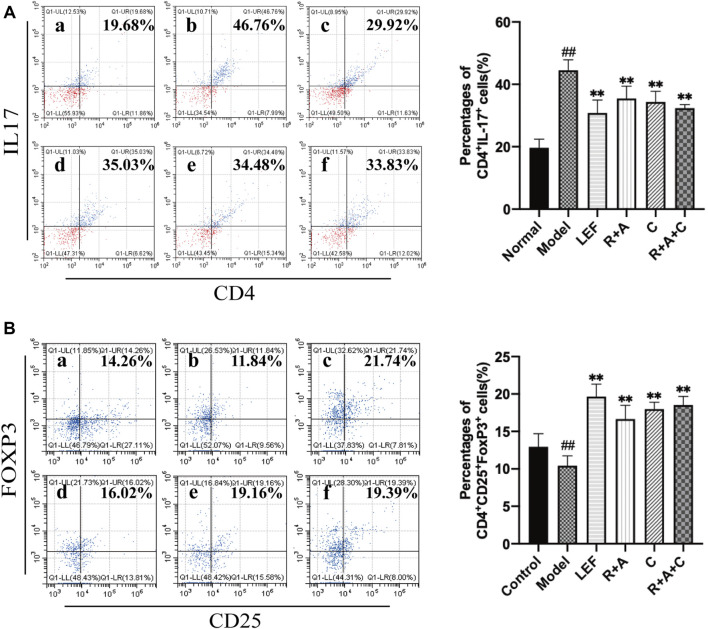
The percentages of Th17 and Treg cells. **(A)** The percentages of CD4^+^IL-17^+^ T-cells. **(B)** The percentages of CD4^+^CD25^+^FOXP3^+^ T-cells. **(a)**, control group; **(b)**, model group; **(c)**, LEF group; **(d)**, *R*. *astragali* + *R*. *angelicae sinensis* (R + A) group; **(e)**, *C*. *lonicerae* (C) group; and **(f)**, *R*. *astragali* + *R*. *angelicae sinensis* + *C*. *lonicerae* (R + A + C) group. The results are presented in the bar charts (*n* = 8). Data are presented as the mean ± SD (*n* = 8); ^#^ *p* < 0.05, ^##^ *p* < 0.01, compared with the normal group; *p < 0.05, **p < 0.01, compared with the model group.

As shown in Figure 5A, the percentages of Th17 (CD4^+^IL-17^+^) cells in the model group was markedly higher than that of the control group (*p* < 0.01). R + A, C, R + A + C, and LEF treatments could downregulate the level of Th17 cells (*p* < 0.01) significantly. As shown in Figure 5B, the percentage of Treg (CD4^+^CD25^+^ Foxp3^+^) cells of model group was lower than that of the control group (*p* < 0.01), and the percentages of Treg cells of the R + A, C, R + A + C, and LEF groups were upregulated significantly (*p* < 0.01). These results indicate that R + A, C, and R + A + C treatment could inhibit the proliferation and differentiation of Th17 and promote the proliferation and differentiation of Treg cells in CIA mice. The effect of R + A + C group was better than those of R + A, and C groups.

### The Proliferation and Differentiation of ILC2s in Synovial Tissue Increased After Herbal Treatments

**FIGURE 6:**
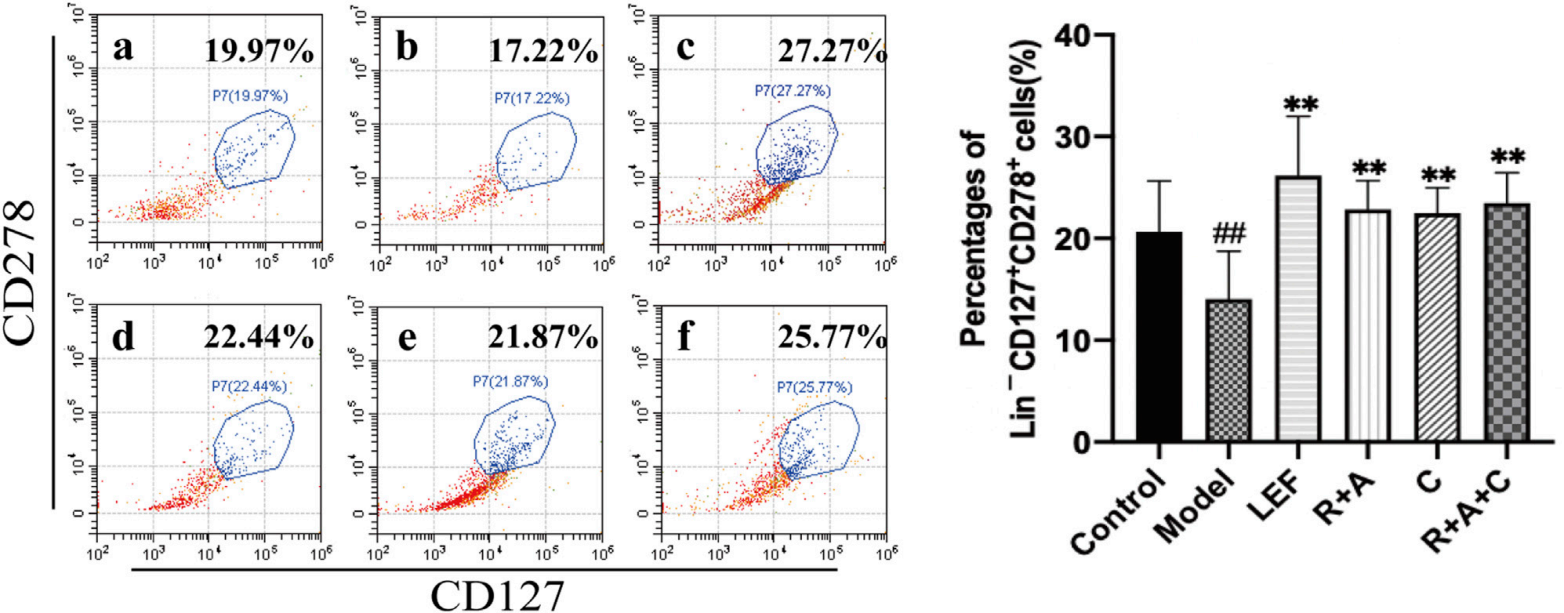
The percentages of Lin^−^CD127^+^CD278^+^ cells. **(a)**, control group; **(b)**, model group; **(c)**, LEF group; **(d)**, *R*. *astragali* + *R*. *angelicae sinensis* (R + A) group; **(e)**, *C*. *lonicerae* (C) group; and **(f)**, *R*. *astragali* + *R*. *angelicae sinensis* + *C*. *lonicerae* (R + A + C) group. The results are presented in the bar charts (*n* = 8). Data are presented as the mean ± SD (*n* = 8); ^#^ *p* < 0.05, ^##^ *p* < 0.01, compared with the normal group; *p < 0.05, **p < 0.01, compared with the model group.

ILC2s could maintain immune homeostasis in the microenvironment of pathological tissue, play an important regulatory role in the activation of Treg cells. Our data showed that the level of ILC2s (Lin^−^CD127^+^CD278^+^) in synovial tissue of mice in the model group was lower than that in the control group (*p* < 0.01). Compared with the model group, the percentages of ILC2s in all treatment groups were increased significantly (*p* < 0.01). The promoting effect of R + A + C group was slightly better than those of the R + A and C groups (Figure 6).

### The Herbal Treatment Induced the Transformation of M1 Macrophages to M2 Subtype in Synovial Tissue

**FIGURE 7:**
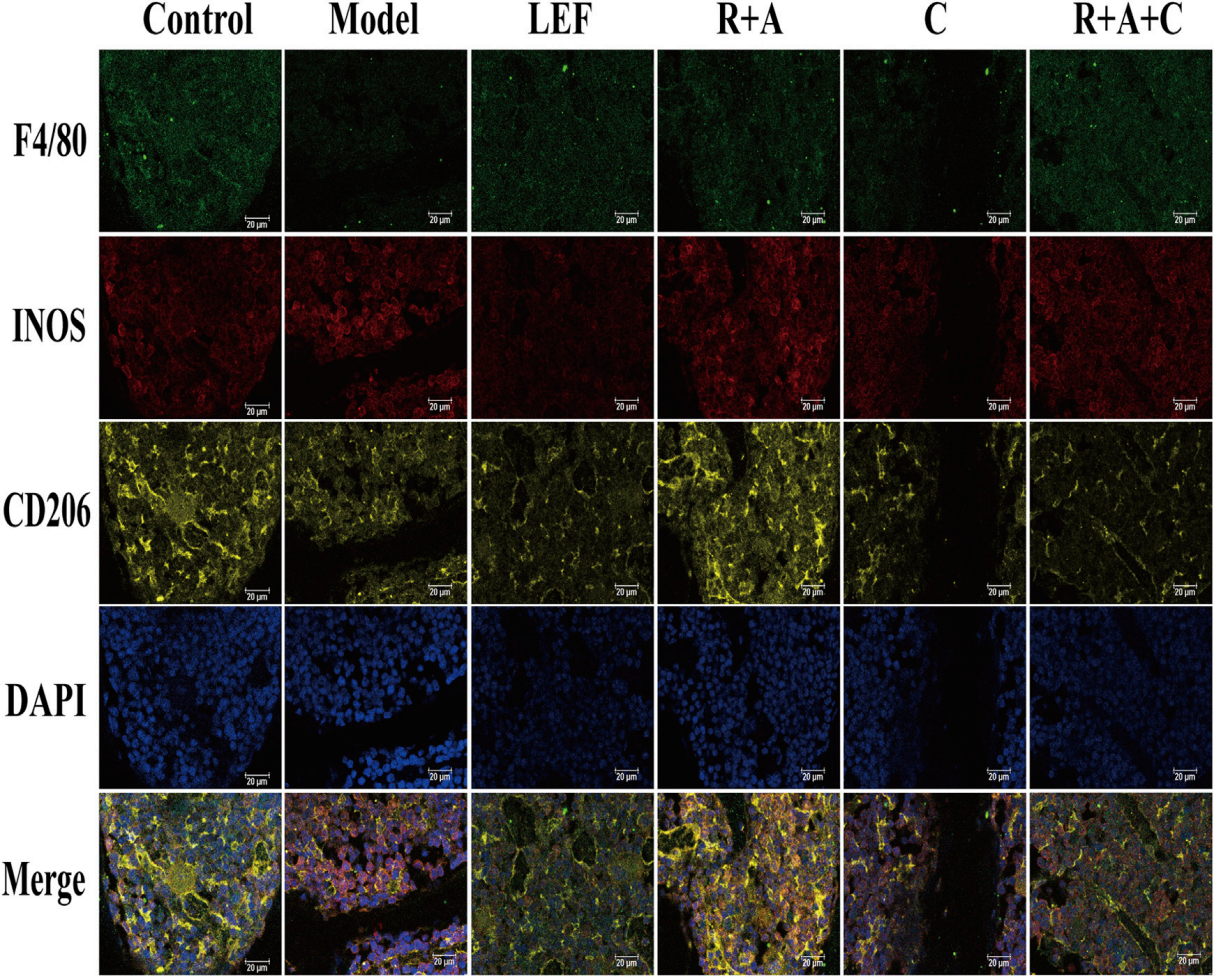
Representative immunofluorescence images of macrophage distribution. Three types of fluorescence labeled M1 and M2 macrophages: F4/80^+^INOS^+^CD206^−^ cells are M1 macrophages, F4/80^+^INOS^−^CD206^+^ cells are M2 macrophages, and F4/80^+^NOS2^+^CD206^+^ cells are M1 and M2 mixed macrophages. The scale bar corresponds to 20 µm throughout.

F4/80, INOS, and CD206 antibodies were used to stain the macrophages triply in immunofluorescence staining. As displayed in Figure 7, INOS were strongly expressed in CIA model group, CD206 were observably expressed in control and treatment groups. R + A, C, R + A + C, and LEF treatment could promote the transformation of M1 (F4/80^+^INOS^+^CD206^−^) macrophages to M2 (F4/80^+^INOS^−^CD206^+^) macrophages. The inducing effect of R + A + C group was better than those of R + A and C groups. These results demonstrate that the therapeutical effect on inflammation suppression of herbal treatment should be related with the polarization of macrophages in synovial tissue of CIA mice.

### The Productions of Pro-Inflammatory and Anti-Inflammatory Cytokines Were Regulated by Herbal Treatments

**FIGURE 8:**
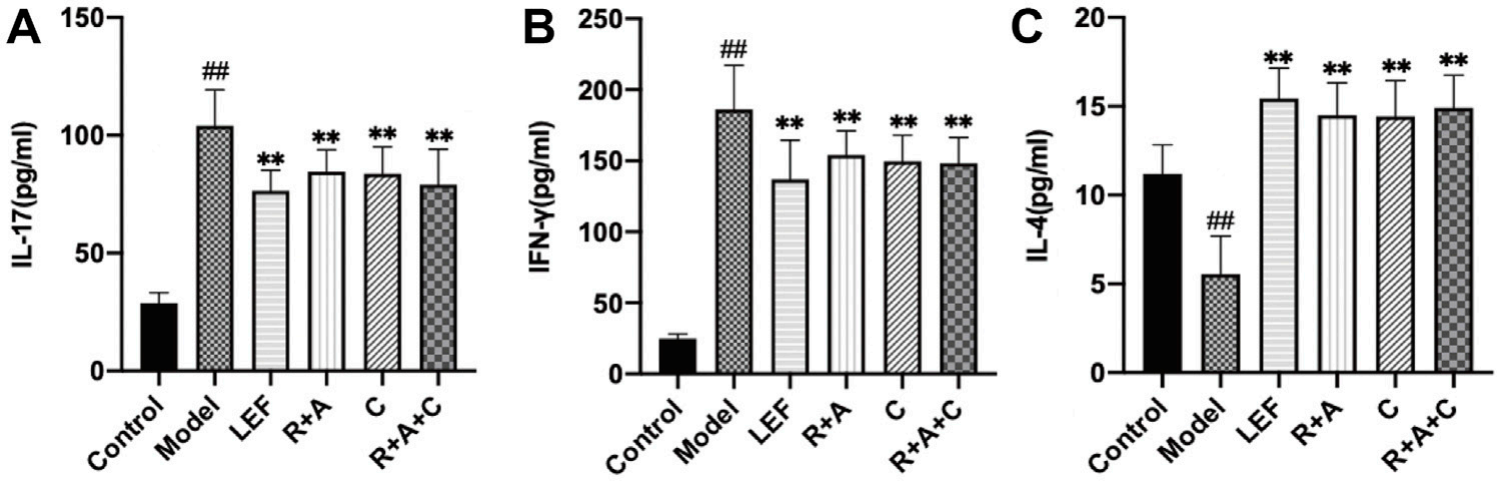
The levels of IL-17, IFN-γ, and IL-4 of control and CIA mice after drug treatment. **(A)** The levels of IL-17. **(B)** The levels of IFN-γ. **(C)** The levels of IL-4. The results are presented in a bar chart. Data are presented as mean ± SD (*n* = 8); ^#^ *p* < 0.05, ^##^ *p* < 0.01, compared with the normal group; *p < 0.05, **p < 0.01, compared with the model group.

As shown in Figure 8, compared with model group, the levels of IL-17 was significantly decreased in R + A, C and R + A + C group (*p* < 0.01). The secretions of IFN-γ were decreased in R + A, C and R + A + C group (*p* < 0.01). The quantities of IL-4 were increased significantly in R + A, C and R + A + C (*p* < 0.01). These results indicated that R + A, C and R + A + C treatment could alleviate synovial inflammation through inhibiting the productions of IL-17 and IFN-γ and increasing IL-4 production. The efficacy of herbal combination of R + A + C was better than those of other herbal treatment.

### The Treatment of Herbal Combination Regulated the Expression Levels of the Key Proteins of JAK/STAT Signaling Pathway

**FIGURE 9:**
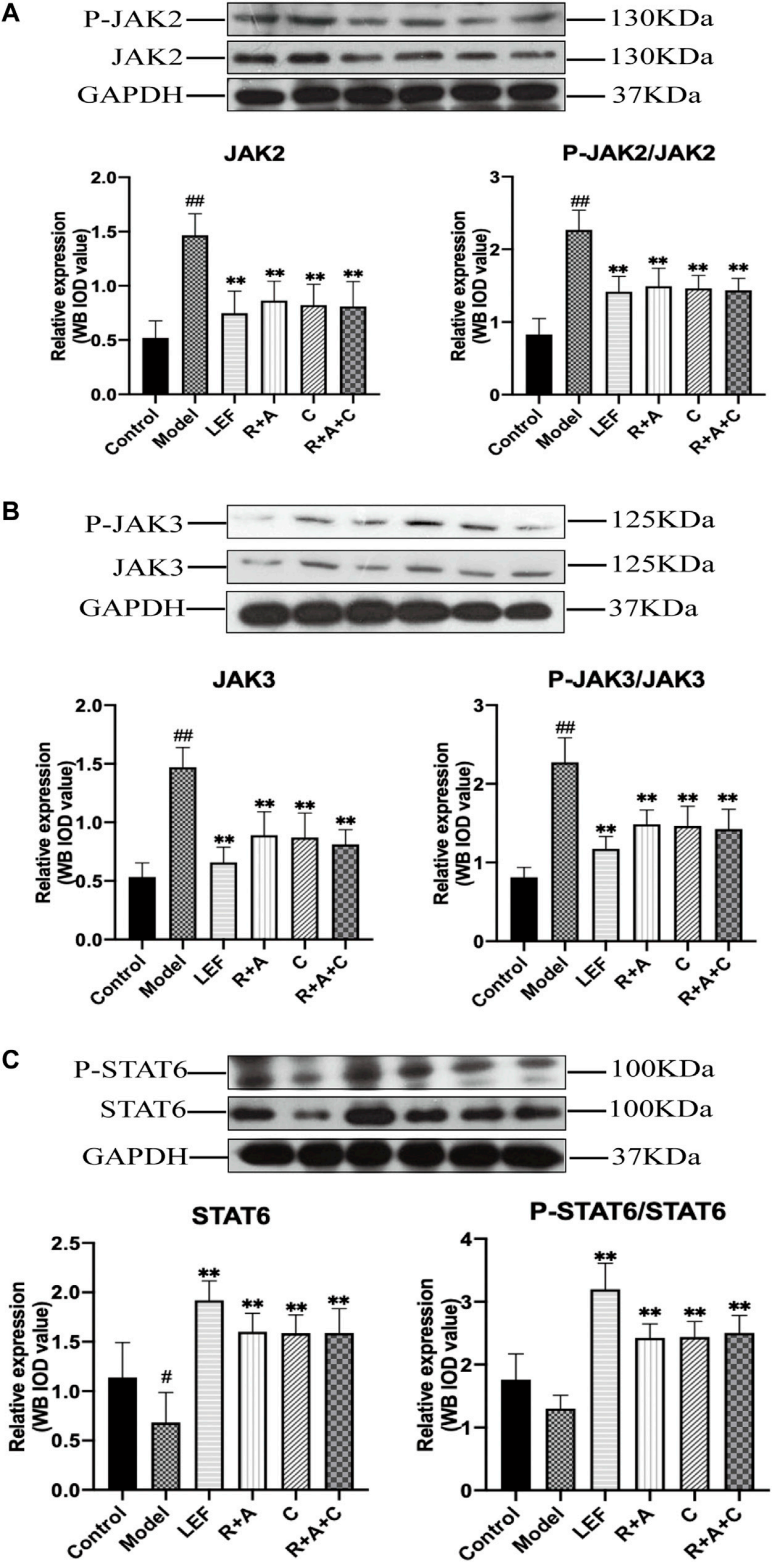
The levels of key molecules in the JAK-STAT signaling pathway of control and CIA mice. **(A)** The protein expression levels of JAK2 and P-JAK2. **(B)** The protein expression levels of JAK3 and P-JAK3. **(C)** The protein expression levels of STAT6 and P-STAT6. Results are presented in a bar chart. The results are presented in a bar chart. Data are presented as mean ± SD (*n* = 3); ^#^ *p* < 0.05, ^##^ *p* < 0.01, compared with the normal group; *p < 0.05, **p < 0.01, compared with the model group.

Western blotting analysis was performed to detect the expression and phosphorylation levels of the key proteins of JAK/STAT signaling pathway in the synovial. Compared with the control group, the protein productions, and phosphorylation levels of JAK2 and JAK3 in synovial tissue of CIA mice in model group were increased significantly (*p* < 0.01). As shown in Figure 9, these abnormally elevated molecular levels were reduced after R + A, C, R + A + C or LEF treatments (*p* < 0.01), and the productions of STAT6 were increased significantly (*p* < 0.01) compared with those of model group. These results indicated that the treatment of herbal combination could alleviate synovial inflammation through inducing the activation of STAT6 signals and inhibiting the expression and phosphorylation of JAK2 and JAK3.

## Discussion

In traditional Chinese medicine (TCM), the herbal formulas consist of several medicinal herbs. The multiple components of herbal combination could act on multiple targets, exert synergistic therapeutic efficacies. In this study, the main components in the aqueous extract of *R*. *astragali*, *R*. *angelicae sinensis*, and *C*. *lonicerae* were analyzed by HPLC-ESI/MS^n^. It has identified seven ingredients in *R*. *astragali*, six ingredients *R*. *angelicae sinensis* and eleven ingredients in *C*. *lonicerae*. Calycosin-7-O-β-glucoside, a bioactive compound isolated from *R*. *astragali*, has biological effects on triggering the processes of bone formation and repair (Park et al., 2021). Moreover, Ononin has been shown to induce apoptosis and reduce inflammation of FLSs in RA (Meng et al., 2021). Calycosin and formononetin, the major compounds of *R*. *astragali*, can regulate the activation of anti-oxidative enzymes (Yu et al., 2009) and promote the osteogenesis of osteoblasts (Kong et al., 2018). Ferulic acid, the main component of *R*. *angelicae sinensis*, has low toxicity and possesses many pharmacological functions including antiinflammatory, antioxidantion, and antimicrobial activity (Zduńska et al., 2018). In addition, vanillic acid can reduce osteoarthritis inflammation and attenuate cartilage degeneration (Huang et al., 2019). Senkyunolide H can attenuate osteoclastogenesis (Yang et al., 2020). The dominant constituents of *C*. *lonicerae* were glycoside, including 3-O-caffeoylquinic acid, loganin, sweroside, secoxyloganin, and so on. Pharmacological studies have confirmed that loganin can ameliorate cartilage degeneration and osteoarthritis development (Hu et al., 2020). Sweroside has been known to promote osteoblast differentiation (Choi et al., 2021). Cynaroside possesses chondroprotective effects (Lee et al., 2020). Therefore, we hypothesis that the herbal combination of *R*. *astragali*, *R*. *angelicae sinensis*, and *C*. *lonicerae* can alleviate synovial inflammation and repair cartilage injury by synergistic effect of multiple components and multiple targets.

TNF and IL-6, as the core hub of RA synovial cytokine network, induce the productions of pro-inflammatory mediators. It can also stimulate the formation of osteoclasts, leading to bone destruction (Choy and Panayi, 2001; Ridgley et al., 2018). Due to the invasion of macrophage like cells and the inflammatory proliferation of FLSs, the synovial lining composed of 1–3 cell layers is significantly thickened. The degree of synovial hyperplasia correlates with the severity of cartilage erosion, leading to the formation of inflammatory pannus, adhesion, and erosion of articular cartilage, resulting in progressive degeneration of articular cartilage (McInnes and Schett, 2011).

Our previous studies have confirmed that the combination of *R*. *astragali* and *R*. *angelicae sinensis* can intervene in T lymphocytes and restore the balance of immune network (Liu et al., 2019). *C*. *lonicerae* can inhibit the inflammatory proliferation of FLSs (Wang et al., 2021). To evaluate the therapeutic effects of the three herbs on RA, we chose the CIA mouse model which mimics the pathological features occurring in human. The histological photographs of CIA mice showed substantial inflammatory cell infiltration, synovial hyperplasia, and cartilage damage in joints. Daily treatment by the combination of these three herbs could alleviate both the ankle swelling and inflammatory cell infiltration, thus the reversals of cartilage damage in the RA-induced histological joint changes.

ILCs are innate immune cells that do not express recombinant antigen receptors and have no antigen specificity (Vivier et al., 2018). ILCs are divided into three different subunits: ILC1s, ILC2s, and ILC3s, according to the cytokines produced by them, have specific nuclear transcription factors and Th cells corresponding to them in the innate immune system (Spits et al., 2013; Eberl et al., 2015). Antigen-specific autoantibodies and autoimmune responsive T-cells activate different types of ILCs through different immune response networks, aggravating or slowing down the inflammatory pathological process of autoimmune diseases (Sonnenberg and Artis, 2015; Exley et al., 2016). ILC1s, coordinating with Th1 cells, secrete IFN-γ, participate in the occurrence and development of autoimmune inflammation in RA (Fang et al., 2020). Th17 cells in the synovial tissues of RA stimulate FLSs through producing large amounts of IL-17, initiating synovial inflammation, and inducing the proliferation and activation of ILC3s. Activation of ILC3s could induce the polarization of monocytes, maintained the persistent inflammation of synovial tissue (Hirota et al., 2018).

However, ILC2 has immunomodulatory and anti-inflammatory effects on the resolution of chronic inflammation in RA (Biton et al., 2016; Omata et al., 2018). Activated ILC2_S_ and Th2 cells both produce powerful anti-arthritis cytokines IL-4 and IL-13 (Bessis et al., 1996; Horsfall et al., 1997). The inhibitory effect of IL-4 is stronger than that of IL-13 on osteoclast differentiation and cartilage destruction (Joosten et al., 1999; Yamada et al., 2007). These cytokines binding to macrophages induce them transforming to a regulatory M2 phenotype through signal transducer and activator of transcription (STAT) 6 activation. M2 macrophages secrete anti-inflammatory effector cytokines such as IL-10 and transforming growth factor (TGF)-β. ILC2s also can produce IL-5 to recruits eosinophils, which shift the balance of macrophages from predominantly being pro-inflammatory M1 macrophages to predominantly being regulatory M2 macrophages, thereby reducing the production of pro-inflammatory cytokines such as TNF and IL-1β by M1 macrophages. Ultimately, this leads to inhibiting the production of pro-inflammatory cytokines and infiltration of pro-inflammatory macrophages and neutrophils in synovium, slowing down the process of synovial inflammation (Chen et al., 2019). In this study, our results showed that the herbal combination of these three herbs could induce the activation of STAT6 signaling pathway stimulated by IL-4. Macrophage polarization played subsequent transformation to anti-inflammatory “M2-like” phenotype, contributing to resolution of synovial inflammation after herbal combination treatment of *R*. *astragali*, *R*. *angelicae sinensis*, and *C*. *lonicerae* treatment. Surprisingly, the therapeutical effect of herbal combination of these three herbs was superior to these of the combination of *R*. *astragali* and *R*. *angelicae sinensis* or *C*. *lonicerae* alone.

Moreover, IL-9 induces the binding of glucocorticoid-induced TNFR-related protein (GITR) ligand (GITRL) to GITR and inducible co-stimulator (ICOS) ligand (ICOSL) to ICOS, increases the proliferation of ILC2s, and then activates the proliferation and differentiation of Treg cells in pathological synovial tissue. Treg cells inhibit the secretion of IL-17 and the proliferation of Th17 cells, promote the resolution of inflammation, repair the inflammatory damage of articular cartilage (Rauber et al., 2017; Chen et al., 2019). We quantified the percentages of Th17 and Treg cells in lymphocytes after the herbal treatment by flow cytometry. The results showed that the combination of *R*. *astragali*, *R*. *angelicae Sinensis*, and *C*. *lonicerae* could inhibit the proliferation and differentiation of Th17 cells, promote the proliferation and differentiation of Treg cells.

Furthermore, abnormal activation of JAK-STAT signaling in RA synovial joints results in the elevated level of matrix metalloproteinase gene expression, increased frequency of apoptotic chondrocytes and prominent “apoptosis resistance” in the inflamed synovial tissue, contributing to progressive degeneration of articular cartilage (Malemud, 2018). In this study, the results indicated that herbal combination treatment could decrease the protein expression of key molecules of JAK/STAT signaling pathway in synovial tissues, suppress the activation of this signaling pathways. Thus, these data indicate that the inhibitory effects on the activation of JAK/STAT signaling pathways may be an important mechanism of *R*. *astragali*, *R*. *angelicae sinensis*, and *C*. *lonicerae* treatment for the alleviation of synovial inflammation.

Our results showed that the combination of these three herbs could increase the levels of ILC2s cells in synovium tissue of CIA mice and induce the polarization of macrophages. Therefore, the main mechanism of inflammation resolution may be related to promoting the proliferation and differentiation of ILC2s, then producing a powerful anti-arthritis cytokine IL-4, which induces the activation of STAT6 signaling pathway and enabling macrophages to transform from pro-inflammatory M1 type to anti-inflammatory M2 type. The decrease of levels of pro-inflammatory cytokines leads to reduced osteoclast formation, alleviated cartilage destruction and bone erosion. The therapeutical effect of herbal combination of these three herbs was superior to these of the combination of *R*. *astragali* and *R*. *angelicae sinensis* or *C*. *lonicerae* alone.

## Conclusion

Overall, our study confirmed that the herbal combination of *R*. *astragali*, *R*. *angelicae sinensis*, and *C*. *lonicerae* could relieve synovial inflammation by regulating the functions of ILC2s and macrophages. Hence, fostering ILC2s activation may offer a novel therapeutic approach for the resolution of inflammation. Moreover, the herbal combination of these three herbs should be prescribed in TCM as a supplement or alternative drugs for RA treatment.

## Data Availability

The original contributions presented in the study are included in the article/Supplementary Materials, further inquiries can be directed to the corresponding authors.
