# Supplementary material for: The Herbal Combination of Radix astragali, Radix angelicae sinensis, and Caulis lonicerae Regulates the Functions of Type 2 Innate Lymphocytes and Macrophages Contributing to the Resolution of Collagen-Induced Arthritis
Source: Front Pharmacol. 2022 Jul 19;13:964559. doi: 10.3389/fphar.2022.964559 (PMC9343953; doi:10.3389/fphar.2022.964559)
Supplement: Supplementary file 1 [file Table1.DOCX]

https://www.jianguoyun.com/p/DSRWvWMQrIXPChi3n8IEIAA
